# Assessing the Burden of Neglected Tropical Diseases in Low-Income Communities: Challenges and Solutions

**DOI:** 10.3390/v17010029

**Published:** 2024-12-28

**Authors:** Francesco Branda, Abdisalam Yusuf Ali, Giancarlo Ceccarelli, Mattia Albanese, Erica Binetti, Marta Giovanetti, Massimo Ciccozzi, Fabio Scarpa

**Affiliations:** 1 Unit of Medical Statistics and Molecular Epidemiology, Università Campus Bio-Medico di Roma, 00128 Rome, Italy; m.ciccozzi@unicampus.it; 2 School of Public Health, Mount Kenya University, Thika P.O. Box 342-01000, Kenya; salanhajji02@gmail.com; 3 Department of Public Health and Infectious Diseases, University of Rome Sapienza, 00161 Rome, Italy; giancarlo.ceccarelli@uniroma1.it (G.C.); dott.albanese.mattia@gmail.com (M.A.); erica.binetti@uniroma1.it (E.B.); 4 Department of Science and Technologies for Sustainable Development and One Health, Università Campus Bio-Medico di Roma, 00128 Rome, Italy; giovanetti.marta@gmail.com; 5 Instituto René Rachou, Fundação Oswaldo Cruz, Belo Horizonte 30190-002, Brazil; 6 Climate Amplified Diseases and Epidemics (CLIMADE)—CLIMADE Americas, Belo Horizonte 30190-002, Brazil; 7 Department of Biomedical Sciences, University of Sassari, 07100 Sassari, Italy

**Keywords:** neglected tropical diseases, mass drug administration, health infrastructure, socioeconomic impact, disease burden, climate change, co-infection, water, sanitation, and hygiene (WASH), global health initiatives, community health education

## Abstract

Neglected tropical diseases (NTDs) represent a group of chronic and debilitating infections that affect more than one billion people, predominantly in low-income communities with limited health infrastructure. This paper analyzes the factors that perpetuate the burden of NTDs, highlighting how poor health infrastructure, unfavorable socioeconomic conditions and lack of therapeutic resources exacerbate their impact. The effectiveness of current interventions, such as mass drug administration (MDA) programs and improved sanitation, in reducing disease prevalence is examined. In addition, the role of climate change, which alters transmission dynamics and expands affected territories, is discussed as an emerging challenge. The analysis suggests that integrated, multisectoral approaches, including health education and infrastructure interventions, are essential to breaking the cycle of poverty and disease. Although international programs have marked significant progress, achieving elimination targets by 2030 requires sustained commitment, innovation, and increased research capacity in endemic countries.

## 1. Introduction

Neglected tropical diseases (NTDs) represent a group of chronic, debilitating infections that disproportionately affect over one billion people worldwide, predominantly in low-income and resource-constrained regions [1]. Despite their significant burden, NTDs have historically been overshadowed by global health priorities, like HIV, malaria, and tuberculosis, resulting in a gap in resources and attention [2]. This neglect perpetuates a vicious cycle of disease and poverty, particularly in marginalized communities with limited access to healthcare, clean water, and sanitation [3]. Addressing this disparity is critical to achieving equitable health outcomes globally. While the impact of NTDs on physical health is well-documented, less attention has been given to their broader socio-economic consequences, which extend beyond immediate health outcomes. For instance, the approximately 19 million disability-adjusted life years (DALYs) lost annually due to NTDs highlight the profound impact on individual livelihoods and community development [4]. The lack of comprehensive studies focusing on the interconnected socio-economic, environmental, and systemic factors sustaining NTDs is a critical gap this paper aims to address. For example, lymphatic filariasis, which affects more than 120 million people worldwide, causes disfiguring swellings (elephantiasis) that cause physical disability and social stigma [5]. Similarly, schistosomiasis, a parasitic disease caused by waterborne worms, affects more than 200 million people worldwide and is associated with chronic pain, liver and kidney damage, and increased risk of bladder cancer [6]. A significant factor contributing to the persistence of NTDs is their endemicity in areas with limited healthcare infrastructure. In many low-income regions, access to primary healthcare is severely limited due to geographic, financial, and systemic barriers [7]. In addition to access to healthcare, socioeconomic factors play a crucial role in perpetuating the burden of NTDs [8]. Poor sanitation and hygiene, along with a lack of clean water, create environments in which NTDs thrive [9]. The continued exposure of vulnerable populations to these pathogens reinforces the cycle of infection and poverty, as affected individuals often face reduced productivity and earning potential, further exacerbating their economic hardship [10]. In many cases, NTD-infected children exhibit stunted growth, malnutrition, and cognitive deficits, which can have lasting effects on their education and future economic opportunities [11]. In response to these challenges, international efforts have been mobilized to address NTDs. Mass drug administration (MDA) programs have proven to be an effective intervention to control several NTDs, particularly lymphatic filariasis, onchocerciasis, and schistosomiasis. However, despite the success of MDA programs in reducing disease prevalence, sustaining high levels of treatment coverage remains a challenge, particularly in remote or conflict-affected areas [8]. The World Health Organization (WHO), in collaboration with pharmaceutical companies and governments, has led initiatives to provide free or low-cost drugs to endemic countries through large-scale MDA programs [12]. The London Declaration on NTDs, signed in 2012, marked a major breakthrough in global efforts to eliminate or control NTDs by 2020. Although significant progress has been made, many elimination targets have not been met, and WHO’s 2021–2030 roadmap calls for a renewed commitment to integrated, multisectoral approaches that address not only the medical treatment of NTDs but also the social determinants of health that drive their persistence [12].

This manuscript represents a narrative review aimed at providing an in-depth exploration of the persistence of NTDs in low-income settings. The review is structured based on three key criteria: (1) examining the global burden and socioeconomic impact of NTDs, (2) analyzing systemic challenges, including health infrastructure deficits and environmental factors, and (3) synthesizing recent data and interventions, such as MDA, sanitation improvements, and climate-adaptive strategies. These criteria are designed to offer a holistic understanding of the multifaceted challenges posed by NTDs. By critically assessing these dimensions, this review highlights actionable insights and solutions tailored to mitigate the persistence of NTDs. The manuscript also explores how these factors interact within a broader framework of global health inequities, emphasizing the importance of integrated, multisectoral approaches in achieving sustainable outcomes.

This structure provides a comprehensive perspective on the challenges and actionable solutions related to NTDs, with the goal of informing policies and public health initiatives. Specifically, the review delves into the interconnected roles of poverty, education, and health system strengthening as pivotal levers for change. Through this lens, the study not only bridges existing knowledge gaps but also provides practical recommendations for researchers, policymakers, and global health practitioners aiming to combat the burden of NTDs in a resilient and impactful manner. It evaluates the effectiveness of current interventions, such as MDA programs and sanitation initiatives, while exploring innovative, integrated, and multisectoral approaches to address these persistent challenges [5]. By bridging existing knowledge gaps, this study aims to inform policies and strategies to combat NTDs in a sustainable and holistic manner.

## 2. Prevalence and Impact of NTDs in Low-Income Communities

### 2.1. Global Burden and Distribution of NTDs

**Figure 1:**
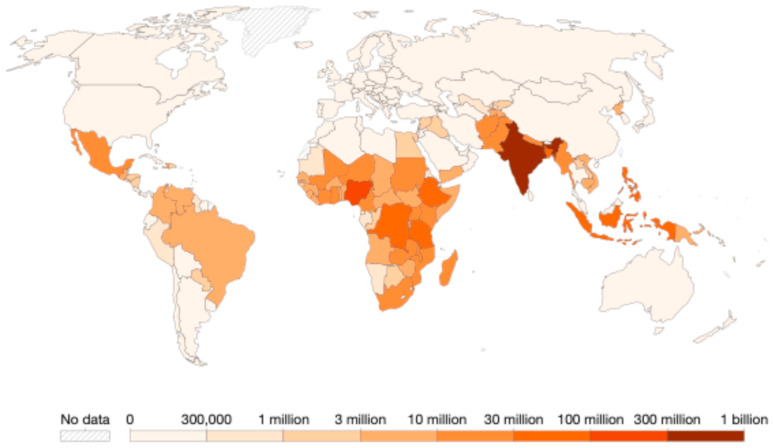
Number of people requiring treatment against neglected tropical diseases, 2021. **Data source:** World Health Organization—Global Health Observatory (2024). Available online: https://ourworldindata.org/grapher/interventions-ntds-sdgs (accessed on 30 October 2024).

The burden of NTDs, typically quantified in DALYs, combines years of life lost (YLL) due to premature mortality and years lived with disability (YLD), offering a comprehensive measure of the impact on mortality and morbidity [13]. Despite substantial progress, with a 26% reduction in people needing NTD interventions since 2010, around 1.62 billion people still required such interventions in 2022. This decrease, while significant, falls short of the road map target for a 90% reduction by 2030, emphasizing the challenges posed by complex and fluctuating health, political, and financial factors. However, there have been milestones worth noting by 2023: WHO recognized 50 countries that have eliminated at least one NTD, halfway to the 2030 goal of 100 countries. Despite these achievements, additional efforts are needed to engage more countries and address a broader range of diseases. The WHO’s introduction of cross-cutting indicators and the Global NTD Annual Reporting Form (GNARF) in 2023 helped highlight ongoing data quality challenges and issues within NTD information systems. The map in Figure 1 illustrates the global distribution of populations requiring treatment for neglected tropical diseases as of 2021. Sourced from WHO data, the map highlights regional disparities, with Africa and Asia bearing the heaviest burdens. India has the largest affected population, with an estimated 100 to 300 million people in need of intervention. Other countries with significant needs include Nigeria, the Democratic Republic of Congo, and Ethiopia, each with populations ranging from 30 to 100 million. This distribution underscores the continued need for targeted interventions in high-burden regions. Between 2016 and 2022, reported deaths from vector-borne NTDs rose by 22%, while integration of NTDs in national health strategies and the development of guidance for disability management related to NTDs improved. Still, the aftereffects of the COVID-19 pandemic remain apparent, with a reduction in people treated through preventive chemotherapy (49 million fewer in 2022 than in 2021) and a drop in integrated treatment coverage. However, this reduction is multifaceted: in some cases, it reflects programmatic successes, such as regions meeting stop-MDA criteria (e.g., millions of treatments halted in Nigeria for onchocerciasis in 2021/2022 due to successful program implementation) [14]. Conversely, other areas may have experienced disruptions influenced by COVID-19’s lingering effects on health systems, logistics, and outreach activities, although earlier pandemic impacts were more pronounced between 2019 and 2020. Additional data are required to disentangle these contributing factors and accurately attribute the observed trends. Access to clean water, sanitation, and hygiene remains limited in NTD-endemic regions, with coverage at 63% among the affected population, and 87.4% are protected from catastrophic health expenses due to NTDs [15].

### 2.2. Co-Infections and Polyparasitism

The epidemiology of NTDs is further complicated by the high prevalence of co-infections and polyparasitism in endemic regions [16,17,18]. Many individuals, particularly in sub-Saharan Africa and Southeast Asia, simultaneously harbor multiple parasitic infections, including soil-transmitted helminths, schistosomes, and filarial worms. A systematic review by Keiser et al. [19] highlighted that polyparasitism is widespread in resource-limited settings and associated with heightened morbidity, particularly in vulnerable populations, such as children and pregnant women.

Hotez et al. [20] emphasize that this phenomenon not only exacerbates the health impacts of individual diseases but also complicates diagnosis, treatment, and public health interventions. For instance, polyparasitism can result in overlapping symptoms, more severe clinical manifestations, and impaired immune responses, making infections harder to treat.

Recognizing NTD polyparasitism in clinical management requires a comprehensive approach, integrating patient history, geographic exposure, and symptoms with laboratory diagnostic tests, including multiplex molecular assays, stool microscopy, and serological tests [21].

Various therapeutic strategies address polyparasitism. For example, since 2008 and 2009, respectively, Senegal has implemented separate, vertical, disease-specific control programs: Seasonal Malaria Chemoprevention and Mass Drug Administration targeting soil-transmitted helminths and schistosomiasis [22,23]. At the same time, integration programs for SMC and MDA for the control of STH and schistosomiasis have also been initiated [19]. This strategy is driven by the potential benefits in terms of cost reduction and improved efficiency in operational planning and the implementation of an integrated malaria and helminth control program in healthcare settings with limited resources, as is often the case in sub-Saharan Africa [24]. Furthermore, integration also has the potential to accelerate progress toward universal health coverage and the achievement of the 2030 agenda [25].

A fascinating aspect of polyparasitism is the potential for one infection to contraindicate the treatment of another. This can arise in various ways:

(a) Drug interactions: treatment for one parasite may negatively interact with treatment for another, reducing efficacy or increasing the risk of adverse effects.
(b) Exacerbation of symptoms: treating one infection could exacerbate the symptoms of another, particularly if the immune response plays a significant role in the pathogenesis of either disease.
(c) Masking of diagnosis: the presence of one parasite could mask the symptoms of another, making diagnosis and appropriate treatment more challenging.
(d) Altered immune response: one infection may alter the host’s immune response, making them more susceptible to a second infection or less responsive to treatment for either.

The complex interplay between multiple parasitic infections within a host highlights the need for a comprehensive approach to diagnosis and treatment. Simply treating individual infections in isolation may not be sufficient and could even be detrimental. A more holistic approach, considering the potential interactions between different parasites, is essential for effective management of polyparasitism.

For example, the co-endemicity of *Loa loa* and *Onchocerca volvulus*, the causative agent of onchocerciasis (river blindness), presents a significant challenge for onchocerciasis control programs. Ivermectin, a key drug in mass drug administration for onchocerciasis, can cause severe adverse events, including encephalopathy, in individuals with high Loa micro-filarial loads [26,27]. These SAEs are thought to be related to the rapid death of *Loa loa* microfilariae following ivermectin treatment, leading to inflammation and potential damage to the central nervous system. This risk necessitates a cautious approach to ivermectin distribution in areas where both parasites are prevalent. Ojurongbe et al. note the public health importance of this co-infection, particularly in regions like southwestern Nigeria [28]. Several strategies have been proposed to mitigate the risk of SAEs:

(a) Test-and-Not-Treat: This strategy involves screening individuals for *Loa loa* microfilaremia before administering ivermectin. Those with high micro-filarial loads are excluded from treatment to avoid the risk of SAEs. Kamgno et al. describes such a strategy implemented in the Okola district [26].
(b) Alternative treatments: Research is ongoing to explore alternative treatments for onchocerciasis in *Loa loa* co-endemic areas. Doxycycline, an antibiotic with anti-filarial properties, has shown promise [29,30].
(c) Improved diagnostics: Accurate and rapid diagnostic tools for both *Loa loa* and *Onchocerca volvulus* are crucial for effective implementation of test-and-treat or alternative treatment strategies.

The presence of *Loa loa* is not the only factor to consider in onchocerciasis treatment. Other factors, such as the intensity of *Onchocerca volvulus* infection and the overall health of the individual, also play a role [25]. The example of *Loa loa* and onchocerciasis underscores the broader importance of considering polyparasitism in the design and implementation of NTD control programs [26]. Failing to account for the interactions between different parasitic infections can lead to unintended consequences and compromise the effectiveness of interventions [26,28,29,30,31,32].

Recognizing polyparasitism in clinical management and public health efforts is critical for developing comprehensive, sustainable solutions to NTD control.

### 2.3. The Impact of Climate Change on NTDs

Climate change is emerging as a critical factor influencing the epidemiology and transmission dynamics of NTDs, particularly vector-borne and waterborne diseases. Rising temperatures, shifting rainfall patterns, and extreme weather events are expected to significantly alter the ecological niches of vectors and parasites (Table 1) [31,32,33,34].

Booth [33] conducted a comprehensive review examining how changes in temperature and precipitation could impact the transmission of NTDs, like dengue, *Leishmaniasis*, and schistosomiasis. The review suggests that warmer temperatures could extend the breeding season of disease vectors, such as mosquitoes and flies, potentially expanding their geographical range into previously non-endemic areas, including higher altitudes and latitudes. This could lead to the exposure of new populations to these diseases. Additionally, increased rainfall and flooding may enhance the breeding habitats for mosquitoes and freshwater snails, further intensifying transmission.

Building on this, a modeling study by Caminade et al. [36] explored the potential changes in the distribution of schistosomiasis in Africa under various climate change scenarios. The study projects that, while certain regions may see a reduction in suitable habitats for schistosome-carrying snails due to temperature extremes, other areas, particularly in East and Southern Africa, could experience an expansion of these habitats. This suggests that climate change may exacerbate existing health inequalities, as some communities face a heightened risk of infection due to environmental changes.

A wide range of studies on the topic highlight the critical need for adaptive public health strategies that not only incorporate climate projections to anticipate and mitigate the impacts of changing ecological conditions on NTD transmission but also emphasize proactive measures. These include vector control, environmental management, and the establishment of robust disease surveillance systems, which will be essential in reducing the future burden of NTDs in the context of a changing climate [31,33,34,35,37].

From a public health perspective, effective prediction of NTD trends and identification of vulnerable populations require a comprehensive understanding of pathogen-vector-host dynamics and the impact of global factors, including climate change, globalization, land use shifts, population growth, migration, and urbanization [31,34]. A One Health framework should steer future research, emphasizing robust data collection on NTDs, their reservoirs, and the drivers of their emergence and re-emergence. Advanced modeling that captures the complexity of NTD life cycles and integrates localized field data is essential. Developing pilot surveillance systems that incorporate multi-sectoral data and actively engage stakeholders in analysis will be key. Such research will provide the evidence base for informed policymaking and the design of One Health strategies for surveillance, detection, and control, addressing both current NTD burdens and future risks of emergence [34].

Among the NTDs that have the greatest impact in low-income settings, five stand out due to their epidemiology and the challenges associated with their control. These diseases are examined below.

#### 2.3.1. Soil-Transmitted Helminth Infections

Soil-transmitted helminth (STH) infections, primarily caused by *Ascaris lumbricoides*, *Trichuris trichiura*, and hookworms (*Necator americanus* and *Ancylostoma duodenale*), remain a significant public health burden, particularly in low-income regions of Africa, Asia, and Latin America, affecting over 1.5 billion people globally [38]. These infections are associated with severe health consequences, including growth stunting, anemia, and impaired cognitive development, contributing to long-term socioeconomic disparities.

**Figure A1:**
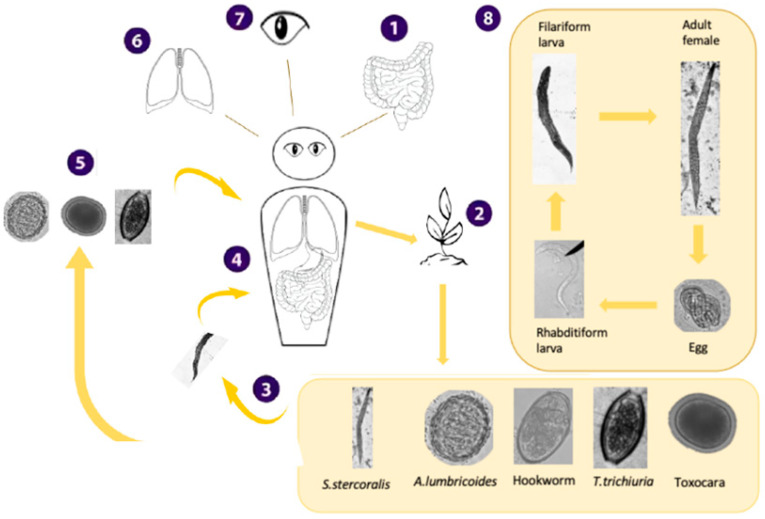
The life cycle of soil-transmitted helminths comprises distinct stages, beginning with adult worms residing in the gastrointestinal tract (GIT). Note. *Ascaris lumbricoides* and hookworms inhabit the small intestine, while *Trichuris trichiura* is found in the large intestine. Mature female worms produce eggs that are passed out of the host’s body through feces (1). In the soil environment, the eggs of *T. trichiura, A. lumbricoides,* and *Toxocara spp.* undergo development but do not hatch. In contrast, hookworm eggs hatch, releasing larvae that develop into the infective L3-stage larvae (2). *Strongyloides stercoralis* larvae hatch within the intestine and are expelled in the feces. These larvae can then either develop into infective larvae or mature into free-living adults (3). Infective hookworm and *S. stercoralis* larvae penetrate the host’s skin, entering the bloodstream and migrating to the lungs before eventually reaching the GIT (4). When *A. lumbricoides, T. trichiura*, and *Toxocara spp*. eggs are ingested by a host, they hatch in the intestines. The resulting larvae may remain in the intestines or migrate to other tissues (5). Hookworm and *A. lumbricoides* larvae migrate from the lungs, travel up the bronchial tree, are swallowed, and ultimately mature in the intestines (6). *Toxocara* larvae, which can accidentally infect humans, may cause toxocariasis by invading various tissues, including the eyes, potentially leading to blindness (7). In cases of autoinfection, *S. stercoralis* larvae can re-enter the host’s body from the gut and spread throughout the system (8). Finally, *S. stercoralis* larvae can also develop into free-living adults that reproduce in the environment, subsequently producing larvae capable of infecting new human hosts.

The lifecycle of soil-transmitted helminths is shown as (Appendix A Figure A1). The symptoms vary depending on the infection’s severity. Light infections often go unnoticed, while more severe infections can lead to abdominal pain, diarrhea, nutrient and blood loss, rectal prolapse, and significant cognitive and physical developmental delays in children [39]. Cognitive impairments, such as difficulties with memory, learning, concentration, and decision-making, are particularly concerning.

##### Epidemiology

The WHO estimates that approximately 1.5 billion people are affected by STHs, with the highest prevalence in tropical and subtropical regions where sanitation is often inadequate [40,41]. A comprehensive study by Pullan et al. [42] provides a detailed global analysis of soil-transmitted helminth infections, estimating that in 2010 819 million people were infected with *A. lumbricoides*, 464.6 million with *T. trichiura*, and 438.9 million with hookworms. The study emphasizes the disproportionate impact on children, with over 270 million preschool-age children and over 600 million school-age children living in areas of intensive transmission. This high prevalence in children is particularly concerning due to the potential long-term effects on growth, cognitive development, and educational outcomes. A more recent systematic review and meta-analysis by Vaz Nery et al. [43] examined the global prevalence of STH infections from 2000 to 2018. They found that, while overall prevalence has decreased, hotspots of high endemicity persist, particularly in sub-Saharan Africa and parts of Asia. The study also highlighted the importance of environmental factors, noting that areas with high rainfall, warm temperatures, and poor sanitation continue to have elevated STH prevalence.

##### The Current Landscape, Achievements, and Challenges in Disease Control

Preventive actions include handwashing before eating or preparing food, thoroughly washing, peeling, and cooking raw fruits and vegetables, avoiding contact with soil and water contaminated by human feces, and wearing shoes to prevent hookworm infection [44]. Diagnosis is confirmed through stool sample analysis, where helminths can be identified microscopically. Treatment requires prescription drugs, such as albendazole or mebendazole, which are effective across helminth species, with treatment duration typically lasting one to three days. The WHO recommends “preventive chemotherapy” for high-risk groups like children, women of childbearing age, and those in high-risk occupations, offering treatment without prior stool examination (https://www.who.int/neglected_diseases/diseases/en/, accessed on 9 November 2024).

Moreover, the WHO has delineated three preventive chemotherapy strategies for controlling soil-transmitted helminth infections. Mass drug administration involves administering preventive chemotherapy to the entire population in endemic regions at regular intervals, irrespective of infection status. Targeted chemotherapy provides anthelmintic treatment to specific high-risk groups, such as those based on age or gender, also at regular intervals and regardless of infection status. Finally, selective chemotherapy offers anthelmintic treatment exclusively to individuals confirmed or suspected to have an STH infection [45].

MDA, treating large groups or entire communities with safe and inexpensive or donated medications, has proven effective in reducing the burden of STH infections [46]. MDA strategies for soil-transmitted helminthiasis vary by country, based on local and national policies [47,48]. The World Health Organization recommends single-dose albendazole (400 mg) or mebendazole (500 mg) for MDA, which are highly effective against *Ascaris lumbricoides* and hookworm but less effective against *Trichuris trichiura* (whipworm). For co-infections with whipworm and hookworm, the WHO advises combining albendazole with ivermectin [49]. MDA strategies for disease control are safe, effective, and cost-effective, costing between US $0.30 and US $0.50 per person treated in most contexts [50]. In any case, the implementation of mass drug administration programs requires significant time, coordination, and financial resources. With the increasing expectation of remuneration for volunteers, the cost-effectiveness of MDA programs needs to be considered. A more expensive, yet more effective drug that requires fewer rounds of MDA may ultimately be more economical for health services compared to a cheaper drug necessitating more frequent administration [51].

#### 2.3.2. Lymphatic Filariasis

**Figure A2:**
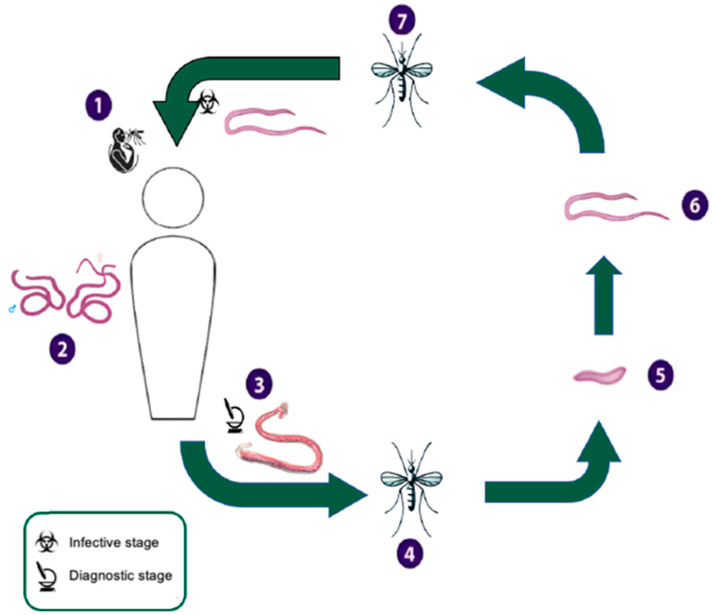
The Brugia malayi and Wuchereria bancrofti Life Cycles in Lymphatic Filariasis. Note. ***Brugia malayi*:** During a blood meal, an infected mosquito (typically of the *Mansonia* or *Aedes* genera) transmits infective, third-stage filarial larvae to a human host. These larvae enter the human body through the bite wound (1), penetrating the skin and migrating to the lymphatic system where they mature into adult worms (2). Adult *B. malayi* worms, although resembling *Wuchereria bancrofti*, are smaller, with females ranging from 43 to 55 mm long and 130 to 170 μm wide, and males measuring 13 to 23 mm in length and 70 to 80 μm in width. These adult worms produce sheathed microfilariae (177–230 μm long and 5–7 μm wide) that exhibit nocturnal periodicity, although *B. malayi* can also exhibit sub-periodicity. (Note that microfilariae are typically absent in *B. pahangi* infections) (3). The microfilariae circulate in the bloodstream after migrating from the lymphatic vessels. When a mosquito takes a subsequent blood meal from an infected human, it ingests these microfilariae (4). Inside the mosquito, the microfilariae shed their sheaths and migrate from the mosquito’s midgut through the proventriculus and cardiac portion to the thoracic muscles. Here, they develop into first-stage larvae (5), then molt into third-stage (6), infective larvae. These infective larvae then travel through the mosquito’s hemocoel to the proboscis (7), where they are positioned for transmission to a new human host during the mosquito’s next blood meal (1). ***Wuchereria bancrofti***: Infective, third-stage *W. bancrofti* larvae are deposited onto the skin of a human host when an infected mosquito takes a blood meal (1). The larvae enter the human body through the bite wound. These larvae then migrate to the lymphatic system where they develop into adult worms (2). Female adult worms are larger than males, measuring 80–100 mm long and 0.24–0.30 mm in diameter, compared to the males’ 40 mm length and 1 mm diameter. The adult worms produce sheathed microfilariae (244–296 μm long and 7.5–10 μm wide) that typically exhibit nocturnal periodicity, although South Pacific strains do not demonstrate this periodicity. These microfilariae actively circulate throughout the lymphatic system and bloodstream (3). A mosquito ingests these microfilariae during a blood meal. Inside the mosquito, the microfilariae shed their protective sheaths and migrate from the midgut, passing through the proventriculus and cardiac region, to the thoracic muscles (4). Within the thoracic muscles, the microfilariae develop into first-stage larvae (5), and then further develop into infective, third-stage larvae (6). These infective larvae then travel through the mosquito’s hemocoel to the proboscis (7), where they are ready to be transmitted to a new human host during the mosquito’s next blood meal (1).

Lymphatic filariasis (LF), commonly known as elephantiasis, is a mosquito-borne disease caused by parasitic filarial nematodes, primarily *Brugia malayi*, *Brugia timori*, and *Wuchereria bancrofti*. The primary hosts for these parasites are humans, with minimal epidemiological significance attributed to animal reservoirs. The typical vectors for *Brugia* species are mosquitoes from the *Mansonia* and *Aedes* genera, while *W. bancrofti* is transmitted by various mosquito species, including *Aedes*, *Anopheles*, *Culex*, and *Mansonia* (https://www.who.int/news-room/fact-sheets/detail/lymphatic-filariasis, accessed on 9 November 2024) [48,49,50,51]. Lifecycle stages of *B. malayi* and *W. bancrofti* are presented in the (Appendix A Figure A2). Clinically, while many LF infections remain asymptomatic, they can lead to severe disfigurement and disability. A hallmark of the disease is the development of severe lymphedema in the limbs, known as “elephantiasis”, which can also affect the genital area (hydrocele) due to lymphatic vessel dysfunction [48,49,50]. Affected limbs may become significantly swollen, with thickened and pitted skin, and are prone to secondary infections due to lymphatic dysfunction. Scrotal hydrocele is commonly observed in infected males. Additional symptoms may include lymphangitis, lymphadenopathy, and eosinophilia during the early stages [52]. A chronic syndrome associated with infections by *W. bancrofti* and *B. malayi*, termed “tropical pulmonary eosinophilia”, involves eosinophilic pulmonary infiltrates, peripheral hyper-eosinophilia, wheezing, chest pain, splenomegaly, and bloody sputum, primarily documented in South and Southeast Asia [51].

Lymphatic filariasis can have severe long-term consequences, with persistent disabilities that endure even after transmission is interrupted. Morbidity Management and Disability Prevention (MMDP) is a critical component of the WHO’s elimination strategies and will be essential for addressing chronic disabilities associated with LF, such as elephantiasis and hydrocele, which continue to impair quality of life even after infection control. Interventions aimed at managing disabilities and preventing complications, such as access to surgical care and rehabilitation, will be crucial for improving long-term outcomes and promoting sustainable impact in reducing LF burden [49,50].

##### Epidemiology

Historically, the geographic distribution of *W. bancrofti* was extensive across tropical regions worldwide, but control measures have since reduced its prevalence. It is now endemic in Sub-Saharan Africa (excluding the southern region), Madagascar, several Western Pacific Island nations, and parts of the Caribbean, with sporadic occurrences noted in South America and Southeast Asia [52]. While India is mentioned here under sporadic occurrences, it is important to note that India bears the highest burden of lymphatic filariasis globally, accounting for nearly two-thirds of the population still in need of MDA as of the Global Programme to Eliminate Lymphatic Filariasis (GPELF) Progress Report 2023 (https://www.who.int/publications/i/item/who-wer-9940-565-576, accessed on 9 November 2024). In contrast, *Brugia* species have a more limited geographical distribution, primarily found in Southeast Asia, with *B. timori* restricted to the Lesser Sunda Islands of Indonesia [53]. Globally, lymphatic filariasis infections decreased from an estimated 199 million (95% UI: 174–234 million) in 2000 to 51 million (95% UI: 43–63 million) in 2018 [53]. While widespread declines in prevalence are evident, some areas within Africa and Southeast Asia remain below proposed elimination thresholds. According to the GPELF, approximately 863 million people in 50 countries remained at risk of LF infection as outlined in the latest progress report (2023), reflecting updated data on the global burden and progress in elimination efforts.

##### The Current Landscape, Achievements, and Challenges in Disease Control

The WHO recommends mass drug administration of anti-helminthic medications targeting at least 65% of the population in endemic areas for 5–7 consecutive years to eliminate lymphatic filariasis as a public health concern [53]. This annual or biannual treatment aims to reduce microfilaremia and antigenaemia, ultimately interrupting transmission [54]. Recommended oral regimens include albendazole alone, or combined with ivermectin, diethylcarbamazine citrate, or both, depending on the specific setting [53]. These medications are typically delivered through public health campaigns, though DEC-fortified salt may be used in certain areas.

While 17 countries have successfully eliminated LF as a public health problem, significant challenges persist in many endemic areas. A comprehensive review by Koudou et al. [55] examined the progress and challenges in LF elimination efforts. They noted that, while MDA programs have been successful in many areas, factors such as systematic non-compliance, migration, and challenges in reaching remote populations continue to hinder elimination efforts in some regions. The economic impact of LF is substantial. A study by Stillwaggon et al. [56] estimated the economic burden of LF in India, finding that the disease results in productivity losses of US $1.5 billion annually, highlighting the broader socioeconomic implications of this NTD.

#### 2.3.3. Schistosomiasis

Schistosomiasis, caused by parasitic flatworms of the genus *Schistosoma*, is widespread in low-income areas, especially in sub-Saharan Africa) [54,55]. Various animals serve as reservoirs for different species: *S. japonicum* affects cattle, dogs, cats, pigs, and others, while *S. mekongi* primarily infects dogs. *S. mansoni* can be found in wild primates but is mainly a human parasite. Snails act as intermediate hosts: *Biomphalaria* for *S. mansoni*, *Oncomelania* for *S. japonicum*, and *Bulinus* for *S. haematobium*, *S. intercalatum*, and *S. guineensis*. *Neotricula aperta* is the sole host for *S. mekongi* (https://www.who.int/news-room/fact-sheets/detail/schistosomiasis, accessed on 9 November 2024) [54,55].

**Figure A3:**
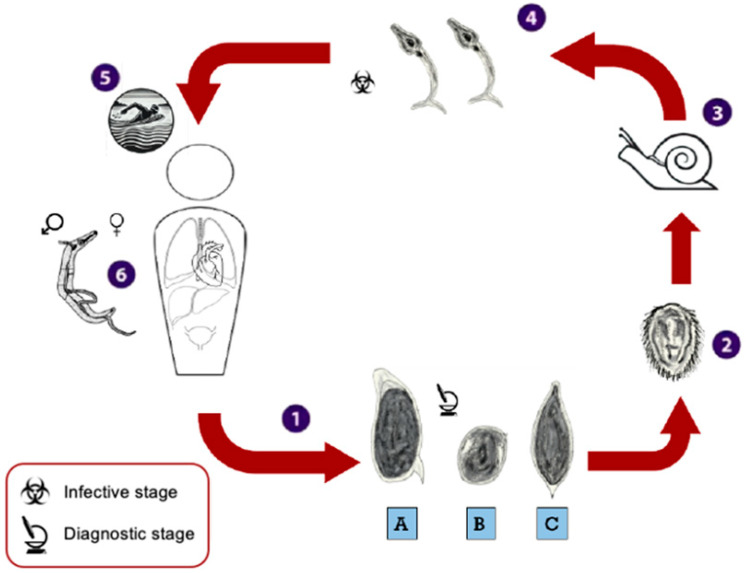
The life cycle of Schistosoma. Note. Schistosome eggs, excreted in either feces or urine depending on the species (1), hatch into free-swimming miracidia under appropriate environmental conditions (2). These miracidia then seek out and penetrate specific snail intermediate hosts (3). Within the snail, the parasite undergoes asexual reproduction, developing through two sporocyst generations and ultimately producing cercariae (4). These cercariae are released from the snail into the surrounding water. Upon encountering a human host, the cercariae penetrate the skin (5), losing their forked tails in the process and transforming into schistosomulae. The schistosomulae migrate through the circulatory system, traveling via the veins to the lungs and heart, and subsequently developing in the liver. Mature schistosomulae then exit the liver through the portal venous system. Adult male and female worms pair up and reside in the mesenteric venules, with the specific location varying depending on the Schistosoma species (6). *S. japonicum* is typically found in the superior mesenteric veins supplying the small intestine (B), while *S. mansoni* prefers the inferior mesenteric veins of the large intestine (A), although some crossover can occur. *S. intercalatum* and *S. guineensis* inhabit the lower mesenteric plexus, even lower than *S. mansoni*, and *S. haematobium* resides in the venous plexus of the bladder and pelvis (C), though it can sometimes be found in rectal venules. Female schistosomes (ranging from 7 to 28 mm in length depending on the species) deposit eggs in the small venules of the portal and peri-vesical systems. These eggs then migrate to the intestinal lumen (*S. mansoni*, *S. japonicum*, *S. mekongi*, *S. intercalatum*/*guineensis*) or the lumen of the bladder and ureters (*S. haematobium*) and are subsequently eliminated from the host’s body through feces or urine, respectively, completing the life cycle (1).

The life cycle of *Schistosoma* is shown in the (Appendix A Figure A3), illustrating the stages from egg excretion to the parasite’s maturation and reproductive phases within the human host.

Clinical symptoms of schistosomiasis arise from the immune response to the parasite’s eggs, with many infections being asymptomatic. Some may experience itchy skin lesions after cercariae penetration) [52]. Acute schistosomiasis (Katayama fever), primarily linked to *S. mansoni* and *S. japonicum*, may cause fever, cough, abdominal pain, diarrhea, hepatosplenomegaly, and eosinophilia. In rare cases, the disease may affect the central nervous system, causing granulomas in the brain (*S. japonicum*) or spinal cord (*S. mansoni*, *S. haematobium*) [57]. Prolonged infections can lead to organ damage, particularly in the liver and spleen due to granulomatous inflammation. *S. mansoni* and *S. japonicum* often result in liver complications, while *S. haematobium* can cause hematuria, tissue scarring, and, rarely, squamous cell carcinoma [58]. The impact of schistosomiasis extends beyond direct health effects. A study by Ezeamama et al. [59] demonstrated the negative impact of schistosomiasis on cognitive function in children, underscoring the long-term developmental consequences of the disease.

##### Epidemiology

Geographically, *S. mansoni* is widespread in sub-Saharan Africa, parts of South America, and the Caribbean, with sporadic reports from the Arabian Peninsula. *S. haematobium* is found in Africa and the Middle East, while *S. japonicum* exists in China, the Philippines, and Sulawesi. Less common species, like *S. mekongi,* are confined to Cambodia and Laos, *S. intercalatum* to Congo, and *S. guineensis* to West Africa [58,59]. Hybrid species have been detected in Corsica, France, and parts of West Africa. According to Lai et al. [60], approximately 240 million people were estimated to be infected with schistosomes globally as of 2012, with more than 90% of cases concentrated in sub-Saharan Africa. Africa accounts for an estimated 85% of global schistosomiasis cases, with prevalence rates in some local populations exceeding 50% [61].

##### The Current Landscape, Achievements, and Challenges in Disease Control

While many schistosomiasis-endemic countries have control programs, and some may have eliminated the disease through improved sanitation and water safety, no international verification guidelines exist [61].

Lai et al. highlighted the significant progress made in some countries through preventive chemotherapy, while noting persistent high-transmission areas in others [59]. Colley et al. [6] report that at least 230 million people require preventive treatment annually, with over 90% of the global burden concentrated in Africa. In 2021, over 251.4 million people required preventive treatment for schistosomiasis, with more than 75.3 million receiving treatment (https://www.who.int/news-room/fact-sheets/detail/schistosomiasis, accessed on 9 November 2024). Schistosomiasis control primarily focuses on reducing disease through periodic, large-scale administration of praziquantel. A more comprehensive approach, incorporating access to safe drinking water, improved sanitation, and snail control measures, would further reduce transmission (https://www.who.int/news-room/fact-sheets/detail/schistosomiasis, accessed on 9 November 2024) [59,60].

Travelers, particularly those engaging in adventure or ecotourism, missionaries, volunteers, and military personnel, face an increased infection risk [62,63]. Outbreaks have occurred among travelers on African river trips. Most travel-related cases originate in sub-Saharan Africa, including popular destinations, like Lake Malawi, Lake Tanganyika, and various rivers. Travelers should be aware that most freshwater sources in Africa are potentially contaminated, regardless of local assurances [61].

#### 2.3.4. Trachoma

Trachoma, caused by repeated infections with *Chlamydia trachomatis* [42], is the leading infectious cause of blindness worldwide. It primarily affects the poorest and most rural regions in Africa, Central and South America, Asia, Australia, and the Middle East, impacting approximately 1.9 million people. Trachoma is responsible for 1.4% of all blindness globally, with Africa remaining the most affected continent. The infection is spread via direct or indirect contact with eye and nose discharges of infected individuals, particularly children, who serve as the primary reservoir for the bacteria. Additionally, certain species of flies can transmit the disease by meeting these discharges and spreading them to others [64]. Active (inflammatory) trachoma is most common among preschool-aged children in endemic areas, with prevalence rates as high as 60–90%. While an individual’s immune system can clear a single infection, repeated re-acquisition of the bacterium is common, especially in households with proximity to infected individuals. Repeated infections lead to the scarring of the eyelids, known as trachomatous conjunctival scarring, which causes the eyelashes to turn inward and rub against the eye (trachomatous trichiasis). This condition results in severe pain, light intolerance, and corneal scarring, which can ultimately cause irreversible blindness. The age at which visual impairment occurs varies with local transmission intensity though, in highly endemic areas, blindness can develop as early as childhood [65]. However, it is more typical for the onset of blindness to occur between the ages of 30 and 40. Women are disproportionately affected by trachoma, being blinded up to four times as often as men. This disparity is largely due to their more frequent contact with infected children, leading to higher rates of infection. Environmental factors that contribute to more intense transmission of *C. trachomatis* include inadequate hygiene, crowded living conditions, and insufficient access to water and sanitation [65].

##### Epidemiology

As of April 2024, trachoma remains a public health issue in 38 countries, with approximately 1.9 million individuals affected by blindness or visual impairment in endemic areas (https://www.who.int/news-room/fact-sheets/detail/trachoma, accessed on 9 November 2024) [64,65,66].

The Global Trachoma Mapping Project, described by Solomon et al. [65], has provided unprecedented detail on trachoma distribution, revealing persistent hotspots of high prevalence in areas like Ethiopia and South Sudan. In some of these areas, the prevalence of trachoma remains alarmingly high despite global efforts to combat it) [64,67].

##### The Current Landscape, Achievements, and Challenges in Disease Control

The disease disproportionately affects the world’s poorest populations, further entrenching poverty. The economic costs of lost productivity due to blindness and visual impairment are estimated to be between US $2.9 and US $5.3 billion annually. When complications like trichiasis are included, the economic impact rises to US $8 billion) [68].

Despite the enormous socioeconomic burden, significant progress has been achieved: as of October 2024, updated data confirm that 20 countries, including Benin, Cambodia, and Ghana, have been validated by the WHO for eliminating trachoma as a public health problem [68]. This progress reflects the success of the WHO-endorsed SAFE strategy (Surgery, Antibiotics, Facial cleanliness, and Environmental improvement), a comprehensive approach aimed at eliminating trachoma. It includes surgery to treat trachomatous trichiasis, antibiotics (mass administration of azithromycin), facial cleanliness to reduce transmission, and environmental improvement (better water and sanitation) to prevent reinfection [68,69].

Recent data from WHO reported that in 2023, 130,746 people with trachomatous trichiasis underwent surgery, while 32.9 million people were treated with antibiotics. In 2019, before the disruption caused by COVID-19, over 95.2 million people were treated, reflecting the scale of these elimination efforts (https://www.who.int/news-room/fact-sheets/detail/trachoma, accessed on 9 November 2024) [68].

Despite this progress, significant challenges persist; achieving sustainable behavior change and improving environmental conditions remain critical obstacles, particularly in the most affected communities.

The WHO adopted the SAFE strategy in 1993 and subsequently established the WHO Alliance for the Global Elimination of Trachoma by 2020 in 1996 (https://iris.who.int/handle/10665/343635, accessed on 9 November 2024). This Alliance has provided ongoing support to countries in implementing the SAFE strategy and bolstering national capacities through epidemiological surveys, monitoring, and resource mobilization [65,66,67,68,69]. Although the global target for trachoma elimination by 2020 was not met, the neglected tropical diseases roadmap for 2021–2030, endorsed by the World Health Assembly, has set a new target date of 2030 (https://www.who.int/publications/i/item/9789240010352, accessed on 9 November 2024). Accelerated implementation of the SAFE strategy and the full engagement of multiple stakeholders, especially those involved in water, sanitation, and socioeconomic development, remain crucial for achieving this goal [65,66,67,68,69].

#### 2.3.5. Onchocerciasis

**Figure A4:**
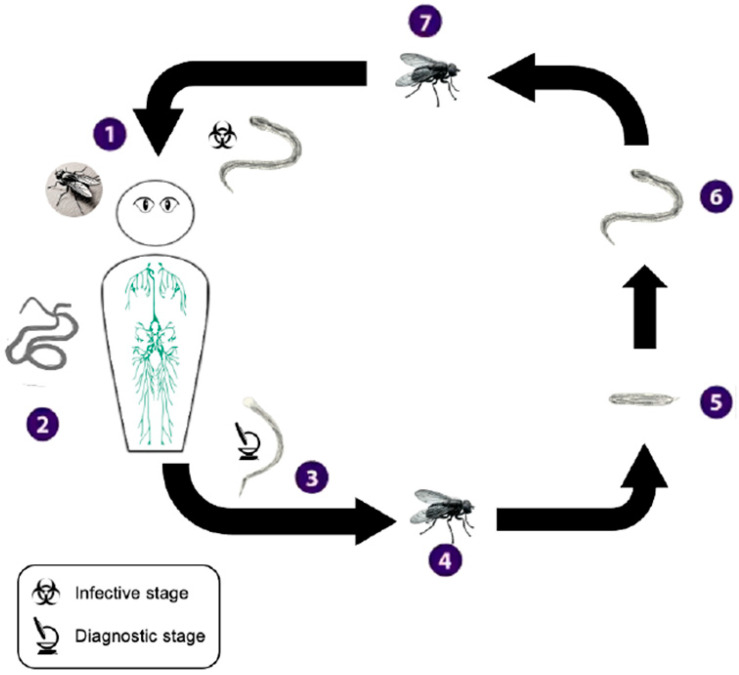
The *Onchocerca volvulus* Life Cycle. Note. Infective, third-stage *O. volvulus* larvae are transmitted to a human host when an infected black fly (genus *Simulium*) takes a blood meal (1). The larvae enter the human body through the bite wound and migrate to the subcutaneous tissues. In these tissues, the larvae develop into adult filariae (2), typically residing within subcutaneous nodules. Adult worms can live within these nodules for approximately 15 years, with each nodule potentially containing multiple male and female worms. Female worms are considerably larger than males, measuring 33–50 cm in length and 270–400 µm in diameter, compared to the males’ 19–42 mm length and 130–210 µm diameter. Within the nodules, female worms produce sheathless microfilariae (220–360 µm long and 5–9 µm in diameter) for up to 9 years. These microfilariae (3) can survive for up to 2 years and are primarily found in the skin and lymphatic vessels of connective tissues, although they can occasionally be detected in peripheral blood, urine, and sputum. A black fly ingests the microfilariae during a blood meal (4). Once ingested, the microfilariae migrate from the black fly’s midgut, through the hemocoel, to the thoracic muscles. Here, they develop into first-stage larvae (5), and subsequently into infective, third-stage larvae (6). These infective larvae then migrate to the black fly’s proboscis (7), where they are ready to infect a new human host during the fly’s next blood meal.

Onchocerciasis, commonly known as river blindness, is a devastating parasitic disease caused by the nematode worm *Onchocerca volvulus*. It is considered one of the most severe NTDs, with 99.7% of cases occurring in sub-Saharan Africa, although it also occurs in certain regions of Latin America and Yemen [69]. The disease is transmitted through the bites of infected blackflies that breed near fast-flowing rivers. The life cycle of *O. volvulus* is illustrated in (Appendix A Figure A4), highlighting the various stages of the parasite from larval inoculation to maturation and production of microfilariae within the human body [69,70]. As the infection progresses, the parasitic worms can spread to the eyes, leading to visual impairment and ultimately, permanent blindness (https://www.paho.org/en/topics/onchocerciasis-river-blindness, accessed on 9 November 2024) [68,69].

##### Epidemiology

Recent data indicate that more than 220 million people reside in areas where onchocerciasis is actively transmitted. It is estimated that onchocerciasis is responsible for blindness or severe vision loss in approximately 1.5 million people worldwide, primarily in sub-Saharan Africa [69], resulting in 46,000 new cases of blindness each year, leaving 270,000 individuals blind and an additional 500,000 visually impaired [68,69]. Onchocerciasis predominantly affects 37 countries, with 30 located in sub-Saharan Africa, spanning from Senegal to Ethiopia and Angola to Tanzania, along with smaller foci in Sudan and Yemen [70]. While formerly endemic in localized areas of Brazil, Colombia, Ecuador, Guatemala, Mexico, and Venezuela, targeted control efforts have reduced its presence in Latin America. Despite these interventions, onchocerciasis currently remains a substantial public health challenge (https://apps.who.int/iris/handle/10665/275724, accessed on 9 November 2024) [68,69].

##### The Current Landscape, Achievements, and Challenges in Disease Control

Efforts to control and eliminate onchocerciasis have primarily focused on MDA programs that distribute the antiparasitic medication ivermectin. Mass drug administration with ivermectin, the current standard for onchocerciasis control, targets the immature microfilariae but not the adult worms. Studies have demonstrated the effectiveness of these programs in reducing disease prevalence, with significant progress made in many parts of Africa [71]. However, challenges remain in maintaining long-term control, particularly in areas with high transmission rates and issues with drug compliance [72].

Ongoing research aims to develop new tools and strategies to address the persistent public health threat posed by onchocerciasis. This includes exploring alternative treatment options, improving diagnostic capabilities, and enhancing community-based approaches to increase ivermectin coverage and adherence [73,74].

Additionally, there is a growing focus on integrating onchocerciasis control efforts with those targeting other NTDs to maximize the impact and efficiency of interventions.

For example, in Southwest Cameroon, onchocerciasis prevalence remains higher than expected despite 12 years of community-directed treatment with ivermectin (CDTi). This may be due to the co-endemicity of *Loa loa*, leading to treatment non-adherence due to fear of adverse reactions. Alternative strategies recommended by the WHO include doxycycline or vector control [75]. This research aims to implement and evaluate a test-and-treat strategy using doxycycline, a macrofilaricide that sterilizes adult worms and shortens their lifespan, combined with focal vector control. This approach addresses the limitations of ivermectin and community perceptions [73,74,75]. Biological monitoring will assess the impact on skin microfilariae prevalence, while vector control will involve targeted larvicide application to local rivers and tributaries. Social science methods will investigate risk factors for CDTi ineffectiveness and evaluate the acceptability and feasibility of the alternative strategies [75].

## 3. Challenges

Access to quality healthcare services remains a significant challenge in low-income communities, particularly in regions heavily burdened by NTDs. Barriers to accessing healthcare in these settings are multifaceted, encompassing both systemic and individual-level factors that contribute to poor health outcomes and the perpetuation of poverty. One of the primary barriers to healthcare access in low-income communities is the scarcity of healthcare infrastructure and resources. Many rural and underserved areas lack sufficient healthcare facilities, trained medical personnel, and essential medicines, making it difficult for individuals to receive timely and effective treatment [7]. This inadequacy is further compounded by the geographic isolation of many low-income communities, where the nearest healthcare facility may be several hours’ journey away, often on foot or via unreliable transportation. As a result, individuals are less likely to seek care when needed, leading to delayed diagnoses and worsened health conditions [76]. Economic barriers also play a crucial role in limiting access to healthcare in low-income settings. Even when healthcare services are available, the cost of care, including consultation fees, medication, and transportation, can be prohibitive for individuals living on less than US $2 per day. This financial burden often forces families to make difficult choices between seeking medical care and meeting other basic needs, such as food and shelter [77]. Consequently, many individuals forego necessary treatments, leading to the progression of preventable and treatable diseases. In addition to these structural barriers, a lack of awareness and of health education significantly impedes access to healthcare in low-income communities. Many individuals in these settings may not recognize the symptoms of diseases or understand the importance of seeking medical attention promptly. This lack of health literacy is often exacerbated by low levels of formal education and limited exposure to public health campaigns [78]. Without adequate health education, individuals are less likely to engage in preventive behaviors or adhere to prescribed treatments, further contributing to poor health outcomes [19,20,21,22,23,24,25,26,27]. The lack of awareness is particularly concerning in the context of NTDs, where the symptoms of many diseases are subtle or slow to develop, leading to a delay in seeking care. For instance, the early symptoms of lymphatic filariasis or schistosomiasis might be mistaken for less serious conditions, leading individuals to ignore or self-treat these conditions until they become debilitating [20]. Furthermore, cultural beliefs and practices can also influence healthcare-seeking behavior, with some communities relying on traditional healers or home remedies instead of formal medical care [8]. Addressing these barriers requires a comprehensive approach that includes strengthening healthcare systems, improving the availability and affordability of care, and enhancing health education and awareness. Efforts to expand healthcare access must prioritize the most vulnerable populations, ensuring that they receive the necessary information, resources, and support to manage their health effectively.

### 3.1. Addressing the Lack of Therapeutic Resources for NTDs

The global health landscape is marred by a stark disparity in access to essential medicines, particularly for NTDs, that disproportionately affects the most impoverished and marginalized populations [79]. A fundamental driver of this disparity lies in the misalignment between the priorities of the pharmaceutical market and the needs of neglected communities. NTDs predominantly afflict populations with limited purchasing power, resulting in low profit margins that fail to incentivize significant investment in research and development by pharmaceutical companies. The high costs and inherent risks associated with drug development further deter investment in diseases with limited market potential, creating a vicious cycle, where lack of profitability reinforces neglect [80,81]. This economic reality is compounded by a lack of awareness and political will. NTDs often remain invisible in mainstream media and public discourse, receiving scant attention compared to diseases prevalent in higher-income countries. This lack of visibility translates into inadequate funding for research, hindering the development of new diagnostics, treatments, and control strategies.

Furthermore, many NTD-endemic countries lack the robust research infrastructure and capacity needed to independently conduct clinical trials and develop new treatments. This reliance on external funding and expertise further exacerbates disparities, highlighting the urgent need for capacity building and knowledge transfer to empower endemic countries in their fight against NTDs [81]. The inherent complexity of NTDs presents additional challenges. Encompassing a diverse range of pathogens, each requiring specific treatment approaches, developing drugs for NTDs is inherently complex and demands significant resources. Moreover, the chronic and debilitating nature of many NTDs leads to long-term disability, impacting economic productivity and perpetuating the cycle of poverty.

**Figure 2:**
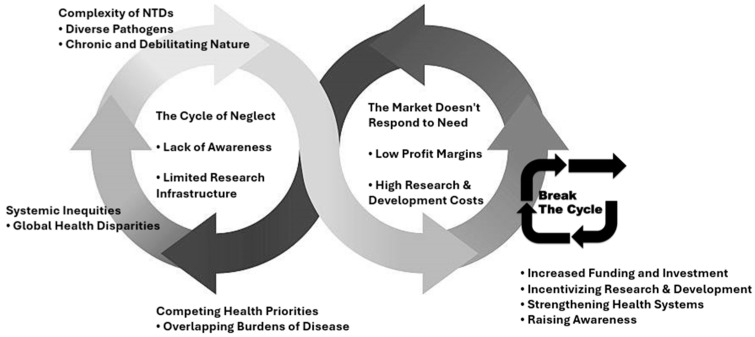
Interconnected factors behind the lack of therapeutic resources for neglected infections.

Addressing this multifaceted crisis necessitates a paradigm shift in global health priorities. Increased and sustained funding from governments, international organizations, and philanthropic foundations is paramount to support research, drug development, and the strengthening of healthcare systems in endemic countries. Innovative financing mechanisms, such as advance market commitments and prize funds, can incentivize private sector investment by mitigating financial risks and aligning incentives with public health needs [82,83]. Equally important is the need to raise public awareness and generate political will. Advocacy efforts must highlight the human and economic toll of NTDs, emphasizing their impact on global development and security. By bringing these diseases to the forefront of global health agendas, we can foster a sense of shared responsibility and mobilize resources to combat this silent crisis (Figure 2).

### 3.2. MDA Programs

Mass Drug Administration programs represent a cornerstone strategy in global health’s ongoing fight against neglected tropical diseases [79,80,81,82,83]. This approach, characterized by the administration of safe and effective medications to entire populations within endemic areas irrespective of individual infection status, presents a powerful tool with the potential to significantly reduce the burden of NTDs [79,80,81,82,83]. The rationale for MDA stems from the high prevalence and transmission rates of these diseases, often exacerbated by inadequate sanitation and hygiene infrastructure in resource-limited settings [79,80,81,82,83].

Furthermore, the asymptomatic nature of many NTD infections necessitates a population-level intervention, like MDA, to effectively disrupt transmission chains. The ease of administration, often through oral medications, and the cost-effectiveness of treating entire populations compared to individual diagnosis and treatment, make MDA a pragmatic approach, particularly in resource-constrained contexts [79,80,81,82,83].

MDA programs have demonstrated remarkable success in controlling and eliminating several NTDs [80,81,82]. Notably, MDA with ivermectin has dramatically reduced the incidence of blindness and skin disease caused by onchocerciasis [84]. Similarly, programs utilizing albendazole and ivermectin or diethylcarbamazine have significantly reduced the disability burden associated with lymphatic filariasis [85]. The mass distribution of azithromycin has also contributed to a substantial decline in trachoma, the leading infectious cause of blindness globally [86,87].

**Figure 3:**
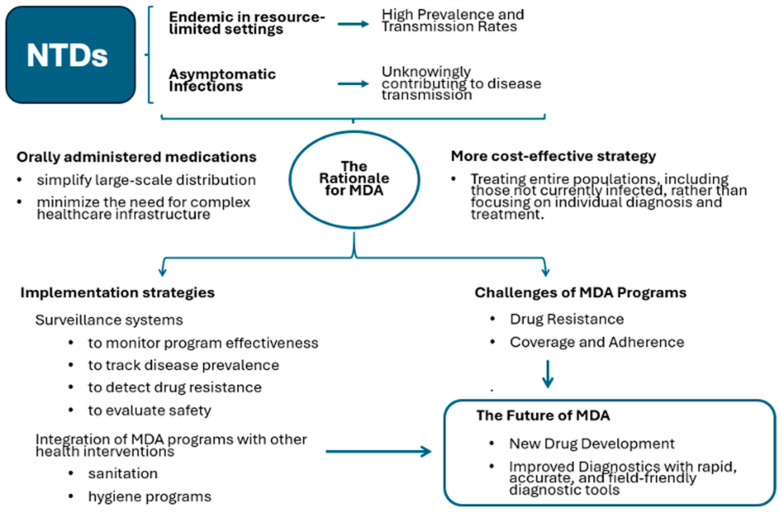
Rationale, challenges and perspectives of MDA.

Despite these successes, challenges remain [88]. The emergence and spread of drug resistance threaten the long-term effectiveness of MDA programs, necessitating ongoing research and development of new medications. Ensuring high treatment coverage and adherence to medication regimens across multiple rounds of MDA can be logistically challenging, particularly in remote or conflict-affected areas [89,90]. Robust surveillance systems are also crucial for monitoring program effectiveness, tracking disease prevalence, and detecting potential drug resistance [91,92] (Figure 3).

The future of MDA hinges on continued innovation and adaptation. Discovering and developing new, safe, and effective medications is paramount to combatting drug resistance. Improved, field-friendly diagnostic tools will enable more targeted treatment and enhance surveillance efforts [81,82,83]. Finally, integrating MDA programs with other health interventions, such as water, sanitation, and hygiene programs, can amplify impact and address underlying risk factors. In conclusion, MDA represents a powerful and cost-effective strategy in the fight against NTDs. By addressing the challenges and investing in research and innovation, we can harness the full potential of MDA to alleviate the burden of these diseases and improve the health and well-being of vulnerable populations worldwide [79,80,81,82,83].

## 4. Solutions

A proactive and multifaceted approach is vital to addressing NTDs. Central to this effort are robust health education and community engagement programs, which serve as the foundation for sustainable public health strategies. These initiatives not only amplify the impact of interventions, such as MDA programs, but also transform communities into active participants in their own health outcomes. By equipping communities with the tools and knowledge necessary to combat NTDs, these solutions effectively target the social and behavioral determinants that perpetuate these diseases, ensuring a comprehensive and lasting impact.

### 4.1. Importance of Health Education in NTD Control

Health education plays a pivotal role in the control of NTDs by increasing awareness about the diseases, their transmission, prevention, and treatment. In many low-income and rural communities, there is a significant lack of understanding about NTDs, which often leads to delays in seeking treatment and the continued spread of infections [93]. Effective health education programs can bridge this knowledge gap by providing accurate information tailored to the cultural and social context of the target population. For example, educational campaigns that focus on the importance of hygiene, safe water practices, and the use of preventive medications can significantly reduce the incidence of diseases such as schistosomiasis and soil-transmitted helminthiasis [20]. Moreover, health education can address misconceptions and stigma associated with NTDs, which are often seen as signs of divine punishment or witchcraft in some communities. By dispelling these myths, education helps reduce discrimination against affected individuals and encourages greater participation in public health interventions [94]. The success of health education initiatives depends on their ability to reach and resonate with the target population. This often requires the use of multiple channels, including schools, community meetings, radio broadcasts, and printed materials, to disseminate information effectively. Additionally, involving local leaders and influencers in the delivery of health messages can enhance credibility and acceptance within the community [95]. Community engagement is a complementary strategy that involves actively involving community members in the planning, implementation, and evaluation of health interventions. This participatory approach is crucial for ensuring that public health programs are culturally sensitive, contextually relevant, and widely accepted [96]. When communities are engaged in the process, they are more likely to take ownership of the initiatives and sustain them over time. In the context of NTD control, community engagement can take many forms, including the training of community health workers, the establishment of village health committees, and the involvement of community members in surveillance and monitoring activities. These efforts not only improve the reach and effectiveness of interventions but also build local capacity and resilience against future health challenges. For instance, the success of onchocerciasis (river blindness) control programs in sub-Saharan Africa has been largely attributed to the involvement of community-directed distributors (CDDs). These volunteers, who are selected by their communities, are responsible for administering ivermectin during MDA campaigns and educating their peers about the disease [97]. The community-driven nature of this approach has led to high treatment coverage and significant reductions in disease prevalence. Community engagement also fosters trust between health authorities and local populations, which is essential for the successful implementation of health interventions. In many regions, mistrust of external actors can hinder participation in public health programs, especially when these programs involve sensitive issues, such as mass drug administration or vaccination [98]. By involving communities in decision-making processes and respecting local knowledge and customs, health programs can overcome these barriers and achieve greater impact.

### 4.2. Challenges and Strategies for Effective Health Education and Community Engagement

While the benefits of health education and community engagement are clear, implementing these strategies effectively can be challenging. One of the primary challenges is ensuring that educational messages are accessible and understandable to all members of the community, including those with low literacy levels or limited access to information. To address this, health programs must use clear, simple language and visual aids that can be easily understood by a wide audience [88,96,99]. Another challenge is sustaining community engagement over the long term, particularly in areas where resources are limited, and health priorities may shift. Maintaining community involvement requires ongoing support, including training, incentives, and recognition of the contributions made by community members [100]. Additionally, health programs must be adaptable and responsive to the changing needs and dynamics of the communities they serve. To enhance the effectiveness of health education and community engagement, it is also important to integrate these strategies with other public health interventions. For example, combining education campaigns with MDA or vector control efforts can create synergies that amplify the impact of each component [20]. Furthermore, fostering partnerships between governments, non-governmental organizations (NGOs), and local communities can leverage resources and expertise to achieve better outcomes.

## 5. Conclusions

NTDs remain a group of chronic, debilitating infections that impact over one billion people, primarily in low-income communities across Africa, Asia, and Latin America. Their burden extends far beyond immediate health impacts, negatively affecting quality of life, limiting educational and employment opportunities, and perpetuating poverty in the most affected regions. Diseases such as schistosomiasis, lymphatic filariasis, and trachoma collectively account for millions of years lived with disability and premature mortality, significantly contributing to global health inequality. Despite international efforts, including the 2012 London Declaration and the WHO Roadmap 2021–2030, progress toward eliminating NTDs has been hindered by persistent challenges. Weak health infrastructure often limits access to widespread treatment delivery, while maintaining high coverage in MDA programs remains particularly difficult in remote or conflict-affected areas. Additionally, environmental and social conditions—such as inadequate sanitation, limited access to safe drinking water, and poor personal hygiene—continue to facilitate disease transmission. Climate change exacerbates these challenges by accelerating the expansion of disease vectors and parasites into new regions. Rising global temperatures and shifting weather patterns are extending transmission seasons and increasing the risk of NTDs in previously unaffected populations. Projections indicate that global warming could intensify these impacts, necessitating more adaptive and flexible responses. Moreover, the lack of robust health information systems in many regions contributes to underreporting and inaccurate data, undermining efforts to monitor disease prevalence, evaluate intervention success, and allocate resources effectively. Strengthening these systems is essential to ensure the success of control programs and to better understand the true burden of NTDs. Effectively addressing NTDs requires an integrated, multi-sectoral approach. Beyond medical treatments, tackling the social and economic conditions that enable these diseases to thrive is critical. Community education programs that emphasize prevention, improved hygiene practices, and active participation in health initiatives can enhance adherence to public health interventions. Investments in water and sanitation infrastructure, alongside initiatives to strengthen local economies, are crucial for breaking the cycle of poverty and disease. Collaboration among governments, non-governmental organizations, the pharmaceutical industry, and local communities has proven effective in reducing the social and economic costs of NTDs through strategies such as free drug distribution and the development of targeted treatments. To achieve the ambitious 2030 elimination targets, sustained resource mobilization, enhanced local research and innovation capacities, and the adoption of new technologies for diagnostics and therapeutic management are essential. These steps will improve the effectiveness and sustainability of interventions, ensuring long-term progress. In conclusion, the elimination of neglected tropical diseases would not only improve the health and well-being of millions but would also represent a pivotal step toward overcoming global health inequalities. With sustained commitment, adequate resources, and innovative solutions, a future free of NTDs can become a reality, enabling social and economic development for the world’s most vulnerable communities. 

## Figures and Tables

**Table 1:** Climate variations and their potential impact on NTDs. Based on (a) Tidman et al. [34]; (b) Pavia et al. [31]; (c) Booth [33]; (d) Acosta-España [35].

Climate Variation	Impact on Environment/Vector/Host	NTD Affected	Impact on Transmission/Distribution	Public Health Implications
Increased temperature	Increased vector breeding rates (e.g., mosquitoes), shorter incubation periods for pathogens	*Plasmodium* spp. (malaria), Dengue, Zika, Chikungunya, *Leishmaniasis* spp. (leishmaniasis)	Increased transmission potential, wider vector distribution, faster disease development	Enhanced vector control, surveillance, rapid diagnostic testing, and public awareness campaigns
Altered Rainfall Patterns	Increased breeding sites for vectors (e.g., stagnant water), changes in intermediate host populations, contamination of water sources	Schistosomiasis, Lymphatic Filariasis, Soil-Transmitted Helminths, Guinea worm disease, waterborne NTDs	Altered transmission dynamics, shifts in disease distribution, increased risk of waterborne outbreaks	Improved water and sanitation infrastructure, targeted interventions based on rainfall patterns, water quality monitoring
Extreme Weather Events (e.g., floods, droughts, heatwaves)	Disruption of sanitation systems, displacement of populations, contamination of water sources, increased stress on individuals and healthcare systems	*Vibrio Cholera*, Typhoid, other waterborne diseases, vector-borne diseases	Increased risk of outbreaks, wider spread of disease, reduced access to healthcare	Disaster preparedness plans, rapid response mechanisms, access to safe water and sanitation, strengthening of healthcare infrastructure
Sea Level Rise	Salinization of freshwater sources, coastal erosion, displacement of populations, loss of arable land	Various NTDs, particularly in coastal regions, foodborne NTDs	Altered disease ecology, potential shifts in disease distribution, food insecurity	Climate change adaptation strategies, relocation of vulnerable communities, food security programs
Ocean Acidification	Changes in marine ecosystems, impacting intermediate hosts or vectors	Foodborne trematodiases	Potential changes in transmission dynamics, impacts on food security	Monitoring of marine ecosystems, sustainable aquaculture practices, food safety regulations
Increased Humidity	Increased vector survival and biting rates	Many vector-borne NTDs, including Dengue, Zika, and Chikungunya	Increased transmission potential	Enhanced vector control measures, public health messaging about protective measures
Changes in wind patterns	Dispersal of vectors and pathogens over wider areas	Various vector-borne NTDs	Potential introduction of diseases to new regions	Surveillance for emerging diseases, vector control measures adapted to wind patterns

## References

[1] Hotez P.J., Fenwick A., Savioli L., Molyneux D.H. (2009). Rescuing the bottom billion through control of neglected tropical diseases. Lancet.

[2] Molyneux D.H., Malecela M.N. (2011). Neglected Tropical Diseases and the Millennium Development Goals-why the other diseases matter: Reality versus rhetoric. Parasites Vectors.

[3] Murray C.J., Vos T., Lozano R., Naghavi M., Flaxman A.D., Michaud C., Ezzati M., Shibuya K., Salomon J.A., Abdalla S. (2012). Disability-adjusted life years (DALYs) for 291 diseases and injuries in 21 regions, 1990–2010: A systematic analysis for the Global Burden of Disease Study 2010. Lancet.

[4] Hotez P.J., Molyneux D.H., Fenwick A., Kumaresan J., Sachs S.E., Sachs J.D., Savioli L. (2007). Control of neglected tropical diseases. N. Engl. J. Med..

[5] Dreyer G., Addiss D., Dreyer P., Noroes J. (2002). Basic lymphoedema management: Treatment and prevention of problems associated with lymphatic filariasis. Int. J. Infect. Dis..

[6] Colley D.G., Bustinduy A.L., Secor W.E., King C.H. (2014). Human schistosomiasis. Lancet.

[7] Peters D.H., Garg A., Bloom G., Walker D.G., Brieger W.R., Hafizur Rahman M. (2008). Poverty and access to health care in developing countries. Ann. N. Y. Acad. Sci..

[8] Molyneux D.H., Savioli L., Engels D. (2017). Neglected tropical diseases: Progress towards addressing the chronic pandemic. Lancet.

[9] Strunz E.C., Addiss D.G., Stocks M.E., Ogden S., Utzinger J., Freeman M.C. (2014). Water, sanitation, hygiene, and soil-transmitted helminth infection: A systematic review and meta-analysis. PLoS Med..

[10] Hotez P.J., Pecoul B., Rijal S., Boehme C., Aksoy S., Malecela M., Tapia-Conyer R., Reeder J.C. (2016). Eliminating the neglected tropical diseases: Translational science and new technologies. PLoS Negl. Trop. Dis..

[11] Miguel E., Kremer M. (2004). Worms: Identifying impacts on education and health in the presence of treatment externalities. Econometrica.

[12] World Health Organization Ending the Neglect to Attain the Sustainable Development Goals: A Road Map for Neglected Tropical Diseases 2021–2030. https://www.who.int/publications/i/item/9789240010352.

[13] WHO Methods and Data Sources for Global Burden of Disease Estimates 2000–2021. https://cdn.who.int/media/docs/default-source/gho-documents/global-health-estimates/ghe2021_daly_methods.pdf?sfvrsn=690b16c3_1.

[14] Sakho F., Badila C.F., Dembele B., Diaby A., Camara A.K., Lamah L., Reid S.D., Weng A., Fuller B.B., Sanchez K.A. (2021). Implementation of mass drug administration for neglected tropical diseases in Guinea during the COVID-19 pandemic. PLoS Negl. Trop. Dis..

[15] Jesudason T. (2024). Global progress report on neglected tropical diseases. Lancet Infect. Dis..

[16] Dawaki S., Al-Mekhlafi H.M., Ithoi I. (2019). The burden and epidemiology of polyparasitism among rural communities in Kano State, Nigeria. Trans. R. Soc. Trop. Med. Hyg..

[17] Pullan R., Brooker S. (2008). The health impact of polyparasitism in humans: Are we under-estimating the burden of parasitic diseases?. Parasitology.

[18] Hürlimann E., Yapi R.B., Houngbedji C.A. (2014). The epidemiology of polyparasitism and implications for morbidity in two rural communities of Côte d’Ivoire. Parasites Vectors.

[19] Keiser J., N’Goran E.K., Traoré M., Lohourignon K.L., Singer B.H., Lengeler C., Tanner M., Utzinger J. (2002). Polyparasitism with Schistosoma mansoni, geohelminths, and intestinal protozoa in rural Côte d’Ivoire. J. Parasitol..

[20] Hotez P.J., Molyneux D.H., Fenwick A., Ottesen E., Ehrlich Sachs S., Sachs J.D. (2006). Incorporating a rapid-impact package for neglected tropical diseases with programs for HIV/AIDS, tuberculosis, and malaria: A comprehensive pro-poor health policy and strategy for the developing world. PLoS Med..

[21] Donohue R.E., Cross Z.K., Michael E. (2019). The extent, nature, and pathogenic consequences of helminth polyparasitism in humans: A meta-analysis. PLoS Negl. Trop. Dis..

[22] World Health Organization (2012). WHO Policy Recommendation on Seasonal Malaria Chemoprevention (SMC) for *Plasmodium falciparum* Malaria Control in Highly Seasonal Transmission Areas of the Sahel Sub-Region in Africa (WHO/HTM/GMP/2012.02). https://apps.who.int/iris/handle/10665/337978.

[23] Afolabi M.O., Diaw A., Sall F.B., Diédhiou A., Seck A., Camara B., Niang D., Manga I.A., Mbaye I., Sougou N.M. (2023). Provider and User Acceptability of Integrated Treatment for the Control of Malaria and Helminths in Saraya, South-Eastern Senegal. Am. J. Trop. Med. Hyg..

[24] Gyapong J.O., Gyapong M., Yellu N., Anakwah K., Amofah G., Bockarie M., Adjei S. (2010). 2010 Integration of control of neglected tropical diseases into health-care systems: Challenges and opportunities. Lancet.

[25] World Health Organization (2017). Integrating Neglected Tropical Diseases into Global Health and Development. https://www.who.int/publications/i/item/9789241565448.

[26] Kamgno J., Pion S.D., Chesnais C.B., Bakalar M.H., D’Ambrosio M.V., Mackenzie C.D., Nana-Djeunga H.C., Gounoue-Kamkumo R., Njitchouang G.R., Nwane P. (2017). A Test-and-Not-Treat Strategy for Onchocerciasis in Loa loa-Endemic Areas. N. Engl. J. Med..

[27] Blok D.J., Kamgno J., Pion S.D., Nana-Djeunga H.C., Niamsi-Emalio Y., Chesnais C.B., Mackenzie C.D., Klion A.D., Fletcher D.A., Nutman T.B. (2021). Feasibility of Onchocerciasis Elimination Using a Test-and-not-treat Strategy in Loa loa Co-endemic Areas. Clin. Infect. Dis..

[28] Ojurongbe O., Akindele A.A., Adeleke M.A., Oyedeji M.O., Adedokun S.A., Ojo J.F., Akinleye C.A., Bolaji O.S., Adefioye O.A., Adeyeba O.A. (2015). Co-endemicity of loiasis and onchocerciasis in rain forest communities in southwestern Nigeria. PLoS Negl. Trop. Dis..

[29] Trotignon G., Dixon R., Atekem K., Senyonjo L., Kamgno J., Biholong D., Jones I., Nditanchou R. (2023). Cost of implementing a doxycycline test-and-treat strategy for onchocerciasis elimination among settled and semi-nomadic groups in Cameroon. PLoS Negl. Trop. Dis..

[30] Nditanchou R., Dixon R., Atekem K., Akongo S., Biholong B., Ayisi F., Nwane P., Wilhelm A., Basnet S., Selby R. (2023). Acceptability of test and treat with doxycycline against Onchocerciasis in an area of persistent transmission in Massangam Health District, Cameroon. PLoS Negl. Trop. Dis..

[31] Pavia G., Branda F., Ciccozzi A., Romano C., Locci C., Azzena I., Pascale N., Marascio N., Quirino A., Gigliotti S. (2024). The issue of climate change and the spread of tropical diseases in Europe and Italy: Vector biology, disease transmission, genome-based monitoring and public health implications. Infect. Dis..

[32] Ceccarelli G., Branda F., Giovanetti M., Ciccozzi M., Scarpa F. (2024). The urgent need for arbovirus surveillance and control following a catastrophic event: The case of the DANA flood event in Valencia. New Microbes New Infect..

[33] Booth M. (2018). Climate Change and the Neglected Tropical Diseases. Adv. Parasitol..

[34] Tidman R., Abela-Ridder B., de Castañeda R.R. (2021). The impact of climate change on neglected tropical diseases: A systematic review. Trans. R. Soc. Trop. Med. Hyg..

[35] Acosta-España J.D., Romero-Alvarez D., Luna C., Rodriguez-Morales A.J. (2024). Infectious disease outbreaks in the wake of natural flood disasters: Global patterns and local implications. Infez. Med..

[36] Caminade C., McIntyre K.M., Jones A.E. (2019). Impact of recent and future climate change on vector-borne diseases. Ann. N. Y. Acad. Sci..

[37] Kelly-Hope L.A., Harding-Esch E.M., Willems J., Ahmed F., Sanders A.M. (2023). Conflict-climate-displacement: A cross-sectional ecological study determining the burden, risk and need for strategies for neglected tropical disease programmes in Africa. BMJ Open.

[38] Villabona-Arenas C.J., de Oliveira J.L., Capra C.d.S., Balarini K., Loureiro M., Fonseca C.R.T.P., 587 Passos S.D., Zanotto P.M.d.A. (2014). Detection of four dengue serotypes suggests rise in hyperendemicity 588 in urban centers of Brazil. PLoS Negl. Trop. Dis..

[39] Jourdan P.M., Lamberton P.H.L., Fenwick A., Addiss D.G. (2018). Soil-transmitted helminth infections. Lancet.

[40] World Health Organization Soil-Transmitted Helminth Infections. https://www.who.int/news-room/fact-sheets/detail/neglected-tropical-diseases.

[41] Chen J., Gong Y., Chen Q., Li S., Zhou Y. (2024). Global burden of soil-transmitted helminth infections, 1990–2021. Infect. Dis. Poverty.

[42] Pullan R.L., Smith J.L., Jasrasaria R., Brooker S.J. (2014). Global numbers of infection and disease burden of soil transmitted helminth infections in 2010. Parasites Vectors.

[43] Vaz Nery S., Pickering A.J., Abate E., Asmare A., Barrett L., Benjamin-Chung J., Bundy D.A., Clasen T., Clements A.C., Colford J.M. (2019). The role of water, sanitation and hygiene interventions in reducing soil-transmitted helminths: Interpreting the evidence and identifying next steps. Parasites Vectors.

[44] Ercumen A., Benjamin-Chung J., Arnold B.F., Lin A., Hubbard A.E., Stewart C. (2019). Effects of water, sanitation, handwashing and nutritional interventions on soil-transmitted helminth infections in young children: A cluster-ransomized controlled trial in rural Bangladesh. PLoS Negl. Trop. Dis..

[45] World Health Organization (2017). Guideline: Preventive Chemotherapy to Control Soil-Transmitted Helminth Infections in at-Risk Population Groups.

[46] de Souza D.K., Dorlo T.P.C. (2018). Safe mass drug administration for neglected tropical diseases. Lancet.

[47] Farrell S.H., Coffeng L.E., Truscott J.E., Werkman M., Toor J., de Vlas S.J. (2018). Investigating the effectiveness of current and modified World Health Organization guidelines for the control of soil-transmitted helminth infections. Clin. Infect. Dis..

[48] World Health Organization (2021). Soil-Transmitted Helminth Infections. https://www.who.int/news-room/fact-sheets/detail/soil-transmitted-helminth-infections.

[49] Speich B., Moser W., Ali S.M., Ame S.M., Albonico M., Hattendori J. (2016). Efficacy and reinfection with soil-transmitted helminths 18-weeks post-treatment with albendazole-ivermectin, albendazole-mebendazole, albendazole-oxantel pamoate and mebendazole. Parasites Vectors.

[50] World Health Organization (2017). Crossing the billion. Lymphatic Filariasis, Onchocerciasis, Schistosomiasis, Soil-Transmitted Helminthiases and Trachoma: Preventive Chemotherapy for Neglected Tropical Diseases.

[51] Chong N.S., Smith S.R., Werkman M., Anderson R.M. (2021). Modelling the ability of mass drug administration to interrupt soil-transmitted helminth transmission: Community-based deworming in Kenya as a case study. PLoS Negl. Trop. Dis..

[52] Newman T.E., Juergens A.L. (2024). Filariasis. StatPearls [Internet].

[53] Cromwell E.A., Schmidt C.A., Kwong K.T., Pigott D.M., Mupfasoni D., Biswas G., Shirude S., Hill E., Donkers K.M., Abdoli A. (2020). Local Burden of Disease 2019 Neglected Tropical Diseases Collaborators. The global distribution of lymphatic filariasis, 2000–2018: A geospatial analysis. Lancet Glob. Health.

[54] Stolk W.A., Swaminathan S., van Oortmarssen G.J., Das P.K., Habbema J.D.F. (2003). Prospects for elimination of Bancroftian filariasis by mass drug treatment in Pondicherry, India: A simulation study. J. Infect. Dis..

[55] Koudou B.G., de Souza D.K., Biritwum N.K., Bougma R., Aboulaye M., Elhassan E., Bush S., Molyneux D.H. (2018). Elimination of lymphatic filariasis in west African urban areas: Is implementation of mass drug administration necessary?. Lancet Infect. Dis..

[56] Stillwaggon E., Sawers L., Rout J., Addiss D., Fox L. (2016). Economic costs and benefits of a community-based lymphedema management program for lymphatic filariasis in Odisha State, India. Am. J. Trop. Med. Hyg..

[57] CDC (2024). About Schistosomiasis. https://www.cdc.gov/schistosomiasis/about/index.html.

[58] Ceccarelli G., d’Ettorre G., Riccardo F., Ceccarelli C., Chiaretti M., Picciarella A., Pacifici L.E., Vullo V. (2013). Urinary schistosomiasis in asylum seekers in Italy: An emergency currently undervalued. J. Immigr. Minor. Health.

[59] Ezeamama A.E., Bustinduy A.L., Nkwata A.K., Martinez L., Pabalan N., Boivin M.J., King C.H. (2018). Cognitive deficits and educational loss in children with schistosome infection—A systematic review and meta-analysis. PLoS Negl. Trop. Dis..

[60] Lai Y.S., Biedermann P., Ekpo U.F., Garba A., Mathieu E., Midzi N., Mwinzi P., N’Goran E.K., Raso G., Assaré R.K. (2015). Spatial distribution of schistosomiasis and treatment needs in sub-Saharan Africa: A systematic review and geostatistical analysis. Lancet Infect. Dis..

[61] Montgomery S., Evan Secor W. (2024). Schistosomiasis-CDC Yellow Book. https://wwwnc.cdc.gov/travel/yellowbook/2024/infections-diseases/schistosomiasis.

[62] Rothe C., Zimmer T., Schunk M., Wallrauch C., Helfrich K., Gültekin F., Bretzel G., Allienne J.F., Boissier J. (2021). Developing Endemicity of Schistosomiasis, Corsica, France. Emerg. Infect. Dis..

[63] Wellinghausen N., Moné H., Mouahid G. (2022). A family cluster of schistosomiasis acquired in Solenzara River, Corsica (France)—Solenzara River is clearly a transmission site for schistosomiasis in Corsica. Parasitol. Res..

[64] Taylor H.R., Burton M.J., Haddad D., West S., Wright H. (2014). Trachoma. Lancet.

[65] Solomon A.W., Burton M.J., Gower E.W., Harding-Esch E.M., Oldenburg C.E., Taylor H.R., Traoré L. (2022). Trachoma. Nat. Rev. Dis. Primers.

[66] Tian L., Wang N.L. (2018). Trachoma control: The SAFE strategy. Int. J. Ophthalmol..

[67] Gordon C.A., Kurscheid J., Jones M.K., Gray D.J., McManus D.P. (2017). Soil-transmitted helminths in tropical Australia and Asia. Trop. Med. Infect. Dis..

[68] Sanders A.M., Dixon R., Stuck L., Kelly M., Woods G., Muheki E.M., Baayenda G., Masika M., Kafanikhale H., Mwingira U. (2021). Evaluation of facial cleanliness and environmental improvement activities: Lessons learned from Malawi, Tanzania, and Uganda. PLoS Negl. Trop. Dis..

[69] World Health Organization (2022). Onchocerciasis. https://www.who.int/news-room/fact-sheets/detail/onchocerciasis.

[70] Frallonardo L., Di Gennaro F., Panico G.G., Novara R., Pallara E., Cotugno S., Guido G., De Vita E., Ricciardi A., Totaro V. (2022). Onchocerciasis: Current knowledge and future goals. Front. Trop. Dis..

[71] Tekle A.H., Zouré H.G., Noma M., Boussinesq M., Coffeng L.E., Stolk W.A., Remme J.H. (2016). Progress towards onchocerciasis elimination in the participating countries of the African Programme for Onchocerciasis Control: Epidemiological evaluation results. Infect. Dis. Poverty.

[72] Senyonjo L., Oye J., Bakajika D., Biholong B., Tekle A., Boakye D., Schmidt E., Elhassan E. (2016). Factors associated with ivermectin non-compliance and its potential role in sustaining Onchocerca volvulus transmission in the west region of Cameroon. PLoS Negl. Trop. Dis..

[73] Boussinesq M., Fobi G., Kuesel A.C. (2018). Alternative treatment strategies to accelerate the elimination of onchocerciasis. Int. Health.

[74] Jacob B., Michael E., Unnasch T.R. (2024). Community-Directed Vector Control to Accelerate Onchocerciasis Elimination. Pathogens.

[75] Wanji S., Nji T.M., Hamill L. (2019). Implementation of test-and-treat with doxycycline and temephos ground larviciding as alternative strategies for accelerating onchocerciasis elimination in an area of loiasis co-endemicity: The COUNTDOWN consortium multi-disciplinary study protocol. Parasites Vectors.

[76] Ensor T., Cooper S. (2004). Overcoming barriers to health service access: Influencing the demand side. Health Policy Plan..

[77] Russell S. (2004). The economic burden of illness for households in developing countries: A review of studies focusing on malaria, tuberculosis, and human immunodeficiency virus/acquired immunodeficiency syndrome. The Intolerable Burden of Malaria II: What’s New. What’s Needed. Am. J. Trop. Med. Hyg..

[78] Kickbusch I., Wait S., Maag D. (2006). Navigating Health: The Role of Health Literacy.

[79] Engels D., Zhou X.N. (2020). Neglected tropical diseases: An effective global response to local poverty-related disease priorities. Infect. Dis. Poverty.

[80] Turner H.C., Stolk W.A., Solomon A.W., King J.D., Montresor A., Molyneux D.H., Toor J. (2021). Are current preventive chemotherapy strategies for controlling and eliminating neglected tropical diseases cost-effective?. BMJ Glob. Health.

[81] Malecela M.N. (2019). Reflections on the decade of the neglected tropical diseases. Int. Health.

[82] Fitzpatrick C., Nwankwo U., Lenk E., de Vlas S.J., Bundy D.A. (2018). An investment case for ending neglected tropical diseases. Major Infectious Diseases.

[83] Rust J., Clark A., Woodgate M., Koch C., Mohammed T., Steinmann P., Krentel A., Torres-Vitolas C.A., Carlin A., Pavluck A. (2022). Innovate to eliminate: A prerequisite in NTD programmes. Int. Health.

[84] Richards F.O., Eigege A., Umaru J., Kahansim B., Adelamo S., Kadimbo J., Danboyi J., Mafuyai H., Saka Y., Noland G.S. (2020). The Interruption of Transmission of Human Onchocerciasis by an Annual Mass Drug Administration Program in Plateau and Nasarawa States, Nigeria. Am. J. Trop. Med. Hyg..

[85] Njenga S.M., Kanyi H., Okoyo C., Githinji E., Mwatele C., Matendechero S.H., Omondi W.P., Gitahi P.N., Owaga C., Onsongo J.K. (2024). Triple-drug therapy with ivermectin, diethylcarbamazine and albendazole for the acceleration of lymphatic filariasis elimination in Kenya: Programmatic implementation and results of the first impact assessment. PLoS Negl. Trop. Dis..

[86] McPherson S., Geleta D., Tafese G., Tafese T., Behaksira S., Solomon H., Oljira B., Miecha H., Gemechu L., Debebe K. (2023). Perceptions and acceptability of co-administered albendazole, ivermectin and azithromycin mass drug administration, among the health workforce and recipient communities in Ethiopia. PLoS Negl. Trop. Dis..

[87] McPherson S., Tafese G., Tafese T., Behaksra S.W., Solomon H., Oljira B., Miecha H., Debebe K.A., Kebede B., Gebre T. (2023). Safety of integrated mass drug administration of azithromycin, albendazole and ivermectin versus standard treatment regimens: A cluster-randomised trial in Ethiopia. EClinicalMedicine.

[88] Macfarlane C.L., Dean L., Thomson R., Garner P. (2019). Community drug distributors for mass drug administration in neglected tropical disease programmes: Systematic review and analysis of policy documents. J. Glob. Health.

[89] Taylor M., Thomas R., Oliver S., Garner P. (2022). Community views on mass drug administration for filariasis: A qualitative evidence synthesis. Cochrane Database Syst. Rev..

[90] Hoefle-Bénard J., Salloch S. (2024). Mass drug administration for neglected tropical disease control and elimination: A systematic review of ethical reasons. BMJ Glob. Health.

[91] Maddren R., Phillips A., Rayment Gomez S., Forbes K., Collyer B.S., Kura K., Anderson R. (2023). Individual longitudinal compliance to neglected tropical disease mass drug administration programmes, a systematic review. PLoS Negl. Trop. Dis..

[92] Konopka J.K., Chatterjee P., LaMontagne C., Brown J. (2022). Environmental impacts of mass drug administration programs: Exposures, risks, and mitigation of antimicrobial resistance. Infect. Dis. Poverty.

[93] Pokharel S., Adhikari B., Johnson T., Cheah P.Y. (2024). Interventions to address antimicrobial resistance: An ethical analysis of key tensions and how they apply in low-income and middle-income countries. BMJ Glob. Health.

[94] Parker M., Allen T.I. (2013). Will mass drug administration eliminate lymphatic filariasis? Evidence from northern coastal Tanzania. J. Biosoc. Sci..

[95] Campbell C., Cornish F. (2010). Towards a “Fourth generation” of approaches to HIV/AIDS management: Creating contexts for effective community mobilisation. AIDS Care.

[96] Rifkin S.B. (2014). Examining the links between community participation and health outcomes: A review of the literature. Health Policy Plan..

[97] Amazigo U.V., Brieger W.R., Katabarwa M., Akogun O., Ntep M., Boatin B., N’doyo J., Noma M., Seketeli A. (2002). The challenges of community-directed treatment with ivermectin (CDTI) within the African Programme for Onchocerciasis Control (APOC). Ann. Trop. Med. Parasitol..

[98] Easterly W. (2015). The trouble with the sustainable development goals. Curr. Hist..

[99] Laverack G., Labonte R. (2000). A planning framework for community empowerment goals within health promotion. Health Policy Plan..

[100] Morgan L.M. (2001). Community participation in health: Perpetual allure, persistent challenge. Health Policy Plan..

